# Comparative profiling of surface-sterilized leaf-associated microbiota and flavor-associated metabolites in two Gougunao tea cultivars across fresh and fermented leaf stages

**DOI:** 10.3389/fnut.2026.1859865

**Published:** 2026-09-01

**Authors:** Jiahao Ai, Junjie Lu, Bojian Wang, Junfeng Jiang, Ying Zhou, Xuan Li, Yusong Cao, Shuaiwen Yin, Zhuangzi Zhong, Xiaojin Liang, Huimin Sun, Xinjun Liao

**Affiliations:** 1 School of Life Sciences, Institute of Modern Agricultural Development, Jinggangshan University, Ji’an, China; 2 Key Laboratory of Jiangxi Province for Biological Invasion and Biosecurity, School of Life Sciences, Jinggangshan University, Ji’an, China; 3 Key Laboratory of Jiangxi Province for Functional Biology and Pollution Control in Red Soil Regions, School of Life Sciences, Jinggangshan University, Ji’an, China; 4 Ji'an Ecological Environment Monitoring Center of Jiangxi Province, Ji’an, China; 5 Jiangxi Tongzhou Green Food Development Co., Ltd., Ji’an, China

**Keywords:** fermented leaves, flavor-associated metabolites, fresh leaves, Gougunao black tea, metabolomics, surface-sterilized leaf-associated microbiota

## Abstract

Black tea quality is governed mainly by endogenous enzymatic reactions during processing, whereas relationships between surface-sterilized leaf-associated microbiota and flavor-related chemical variation remain unclear. Here, differences between two Gougunao tea cultivars and between fresh- and fermented-leaf stages were investigated using 16S rRNA/ITS amplicon sequencing, untargeted LC–MS metabolomics, broadly targeted GC–MS volatile profiling in SIM mode, and sensory evaluation. The two cultivars were compared under the same standardized manufacturing procedure. Multivariate ordination and PERMANOVA showed significant differences in bacterial and fungal assemblages. Community composition differed significantly between G1 and G2 in both kingdoms, whereas a significant processing-stage effect was detected only for fungi. Bacterial communities were dominated by Sphingomonas and Methylobacterium, while fungal profiles were enriched in Dothideomycetes-affiliated genera, particularly Setophoma and Cladosporium. LC–MS and GC–MS analyses revealed differences in volatile and non-volatile features between the two tested cultivars that were evident in fresh leaves and remained detectable after processing. Although PICRUSt2 indicated broadly comparable predicted functional profiles among groups, cross-omics analysis after adjustment for cultivar and processing stage identified a limited set of adjusted genus–feature associations across both analytical platforms. Fungal genera from the same broad taxonomic class showed opposing directional association patterns, indicating taxon-specific rather than uniform class-level covariation. These findings describe microbial–chemical covariation in the two tested Gougunao tea cultivars and provide a basis for further investigation; they should not be generalized as a universal cultivar-dependent pattern, and direct microbial causality cannot be inferred.

## 1 Introduction

Tea is among the most widely consumed beverages worldwide, and variation in manufacturing practice, particularly the extent and conditions of oxidation, strongly determines the chemical composition and sensory characteristics of green, oolong, and black tea products (1). As a fully oxidized tea, black tea is valued for its bright reddish infusion, balanced taste, and complex aromatic profile. Theaflavins and thearubigins make major contributions to liquor color, mouthfeel, and body (2). The tea metabolome from plants to processed products has been comprehensively reviewed (3), while the pharmacological and health-related properties of black and green teas have been summarized elsewhere (4, 5). Metabolomics provides a systems-level framework for characterizing complex chemical variation (6, 7). Metabolomics and related chemical-profiling approaches have been used to characterize geographic and climatic variation in tea metabolites and to relate black-tea chemical composition to infusion color and quality (8, 9).

During black tea manufacture, flavor formation is driven predominantly by endogenous enzymes released from leaf tissues during withering and rolling. Polyphenol oxidase and peroxidase mediate catechin oxidation and subsequent coupling reactions, thereby producing theaflavins, thearubigins, and related compounds that shape liquor color and major taste attributes (1, 2). Aroma-active compounds may also be released or newly formed during processing, including terpenoid alcohols such as linalool and geraniol, which are closely associated with floral and fruity notes (10, 11). Tea aroma development therefore reflects interacting biosynthetic, oxidative, and stress-responsive processes under specific manufacturing conditions. Although these transformations are affected by time, temperature, humidity, and mechanical handling, cultivar background is also a major determinant because it defines the initial reservoir of catechins, amino acids, glycosidically bound aroma precursors, and other substrates that constrain subsequent reaction trajectories. Catechin accumulation and related metabolic traits are influenced by both cultivar and environmental conditions (12).

In addition to endogenous enzyme-dominated chemistry, microbial communities associated with tea leaves may covary with host physiology and flavor-related chemical transformations during processing. Tea plants harbor diverse bacterial and fungal assemblages in different tissues and microhabitats (13, 14). Previous studies have documented microbial succession and microbial–quality relationships during black tea manufacturing (15–17). More recently, bacterial-community profiling, volatile analysis, correlation analysis, and functional prediction were integrated to identify taxa potentially associated with aroma formation in black tea (18), while intervention experiments involving the addition of isolated tea-derived microorganisms provided more direct evidence that selected microorganisms can alter black-tea chemical composition (19). Related microbiome–metabolome studies in Pu-erh and Fuzhuan brick teas have also linked microbial succession with carbohydrate utilization and broader metabolite changes (20, 21). Thus, the contribution of the present study does not lie in the general application of microbiome and metabolome analyses to tea. Microbial associations should also be regarded as complementary to, rather than substitutes for, the endogenous enzymatic and oxidative reactions that dominate black-tea flavor formation.

Gougunao tea is a premium tea from Jiangxi Province, China, and is characterized by sweetness, floral–fruity aroma, and a refreshing mouthfeel. Although it has historically been manufactured mainly as green tea, black tea processing has increasingly been adopted to diversify its product styles. Previous tea metabolomics studies have shown that harvest-related factors can substantially affect tea metabolites and taste quality (22)^,^ while metabolomics combined with sensory evaluation has been used to discriminate Gougunao teas from different geographic origins (23). Against the background of the expanding tea microbiome and multi-omics literature, the unresolved question is not whether microbial and chemical datasets can be combined. Rather, it is whether cultivar-associated microbial and chemical differences remain detectable when two cultivars undergo the same standardized manufacturing procedure. It also remains unclear whether genus–feature associations persist after accounting for microbiome compositionality, cultivar background, and processing stage, and whether such associations provide information that is not apparent from broad community differences or coarse predicted functional categories.

To address these questions, the present study employed a controlled two-cultivar × two-stage design involving fresh and fermented leaves produced under the same standardized black tea manufacturing procedure. Bacterial and fungal communities recovered from surface-sterilized leaf tissues were analyzed together with untargeted LC–MS metabolomics and broadly targeted GC–MS volatile profiling in SIM mode. We first characterized cultivar- and stage-associated differences in microbial-community composition and in volatile and non-volatile chemical features. PICRUSt2-based functional prediction and metabolite-pathway enrichment were used only as broad functional and biochemical context rather than as direct evidence of microbial activity. We then tested whether genus–feature relationships remained detectable after compositional transformation and adjustment for cultivar and processing stage. The principal objective was therefore to distinguish broad group-level differences from more specific taxon-level patterns of microbial–chemical covariation. Because the present design was association-based, it was not intended to establish direct microbial causation. We hypothesized that cultivar-associated microbial and chemical configurations would remain partly detectable after shared processing and that adjusted cross-omics analysis would reveal taxon-specific association patterns not apparent from coarse predicted functional profiles.

## 2 Materials and methods

### 2.1 Experimental materials and sampling

Fresh and visually healthy tea shoots from two cultivars, namely the traditional Gougunao small-leaf landrace (G1) and the improved cultivar Gougunao No. 2 (G2), were collected in Suichuan County, Jiangxi Province, China (26.32°N, 114.52°E; altitude 300–500 m) during the spring tea season of 2025 (April–May). Both cultivars were sampled on the same day using the same one-bud-and-two-leaf plucking standard. For each cultivar, three independent replicate batches were established from separately collected shoots; material from different replicates was not pooled. Each replicate batch was independently subjected to withering, rolling, fermentation, and drying. Harvesting time, shoot standard, transportation, initial handling, and manufacturing conditions were standardized across cultivars because postharvest conditions can substantially alter the non-volatile metabolism of tea leaves (24).

For each replicate batch, an aliquot of fresh leaves was collected before processing and the corresponding fermented/processed leaf sample was collected after completion of the standardized black-tea manufacturing sequence. Sample codes were assigned as follows: G1X and G2X refer to fresh leaves from cultivars G1 and G2, respectively, whereas G1F and G2F indicate the corresponding fermented/processed samples. Each sample was divided for downstream analyses: one portion was freeze-dried and ground for physicochemical and metabolomic analyses, and another portion was used for microbial DNA extraction. Throughout the manuscript, sample names follow the cultivar × stage format. Where microbiome ordination figures retain the original sequencing-folder labels, ‘XY’ corresponds to fresh leaves and ‘Rf’ corresponds to processed leaves, matching the ‘X’ and ‘F’ stages used in the metabolomics sections. Detailed sample-label correspondence is provided in Supplementary Table S3.

### 2.2 Surface sterilization and culture-based procedural check

To reduce interference from surface-associated microorganisms, all sampling tools were sterilized by autoclaving before use. Leaf surfaces were disinfected by sequential immersion in 75% ethanol for 30 s, 2% sodium hypochlorite for 2 min, and 75% ethanol for 30 s, followed by three rinses with sterile water. As a culture-based procedural check, the final rinse water was subjected to plate-culture testing, and no detectable microbial growth was observed under the applied culture conditions.

Because the leaf surfaces were sterilized before DNA extraction, the recovered profiles are referred to throughout the manuscript as surface-sterilized leaf-associated microbiota. This term operationally describes microbial DNA profiles recovered from surface-sterilized leaf material and should not be interpreted as proof of strictly intracellular, resident, or endophytic localization. The final-rinse plate assay detected no culturable microorganisms under the applied conditions but could not exclude residual DNA from tightly adherent surface-associated microorganisms or determine taxon-specific removal efficiency.

### 2.3 Black tea processing protocol

The three replicate batches of each cultivar were processed independently into black tea according to the Jiangxi provincial standard DB36/T 1524-2021 (25). All six replicate batches were subjected separately to the same processing sequence and controlled operating ranges. During withering, temperature was maintained at 20–30 °C and relative humidity at 65 ± 5%, with a spreading density of 0.5–0.75 kg m^−2^. Leaves were turned every 2 h, and withering continued for 8–20 h until moisture content declined to approximately 55–60%. The leaves were then rolled for 60–90 min with gradually increased pressure, and the cell-breakage rate exceeded 85%, in accordance with DB36/T 1524-2021. Rolling has previously been shown to induce marked changes in the volatile profile of Congou black tea (26)^.^

Fermentation was performed in an automated chamber at 25–28 °C under relative humidity of at least 90%. The leaf pile was maintained at a thickness of 8–12 cm. Ventilation was applied for 2–3 min every 30 min, and the material was turned every 1–2 h. Total fermentation time ranged from 3 to 5 h and was terminated when the grassy odor had largely diminished and a slight fruity aroma became perceptible, in accordance with DB36/T 1524-2021. Previous studies have demonstrated that fermentation duration and degree substantially affect the flavor and metabolite profiles of black tea (27, 28).

Drying was conducted in three successive steps: strand shaping at 180 °C for 1–5 min, primary drying at 110–140 °C for approximately 15 min until moisture reached 15–20%, and final drying at 80–90 °C until the final moisture content fell below 6%.

### 2.4 Instruments and reagents

The main instruments used in this study included a nucleic acid quantification analyzer, a PCR thermocycler, a tissue homogenizer, a refrigerated high-speed centrifuge, a UHPLC–Orbitrap high-resolution LC–MS system, and a gel electrophoresis imaging system. All chemicals and reagents were of analytical grade or higher. LC–MS-grade methanol and acetonitrile were used in metabolomic analysis, and L-2-chlorophenylalanine was employed as the internal standard. Agarose electrophoresis was performed using TAE buffer, and DNA concentration was determined using a PicoGreen dsDNA assay kit.

### 2.5 Untargeted LC–MS metabolomics

Frozen leaf material was ground under liquid nitrogen and extracted with prechilled methanol/acetonitrile (1:1, v/v). After vortexing, the extract was incubated at −20 °C for 30 min and then centrifuged at 12,000×*g* for 10 min at 4 °C. The collected supernatants were dried under reduced pressure, reconstituted in 50% methanol containing 5 ppm L-2-chlorophenylalanine, and filtered through a 0.22 μm membrane prior to analysis. Quality-control (QC) samples were prepared by pooling equal aliquots from all study samples, and both QC and blank samples were injected throughout the analytical sequence.

Chromatographic separation was carried out on an ACQUITY UPLC HSS T3 column (2.1 × 100 mm, 1.8 μm) maintained at 40 °C, with a flow rate of 0.4 mL/min. Mobile phase A consisted of water with 0.1% formic acid, whereas mobile phase B consisted of acetonitrile with 0.1% formic acid. The gradient program was set as follows: 0–1 min, 2% B; 1–9 min, 2–98% B; 9–12 min, 98% B; 12–12.1 min, 98–2% B; and 12.1–15 min, 2% B.

Mass spectrometric detection was performed using an Orbitrap Exploris 120 equipped with an electrospray ionization source. Data were acquired separately in positive- and negative-electrospray ionization modes. The spray voltages were set to +3.5 and −3.0 kV, the capillary temperature was 320 °C, the sheath gas was 40, and the auxiliary gas was 10. Full-scan MS^1^ data were collected over m/z 70–1,000 at a resolution of 60,000, and MS^2^ data were acquired in DDA Top10 mode at a resolution of 15,000 with an HCD collision energy of 30%.

Raw LC–MS data acquired in positive- and negative-ion modes were processed independently using XCMS for peak detection, retention-time correction, peak alignment, and feature-table construction (29), resulting in polarity-specific feature tables. Peak intensities were normalized to the internal standard and total ion current, followed by Pareto scaling. Features showing poor QC reproducibility (QC RSD > 30%) were discarded, and missing values were imputed using the half-minimum method. For integrated downstream analyses, QC-qualified annotated features retained from the two polarity-specific tables were combined into a non-redundant feature matrix, while the ionization mode of each retained feature was preserved. When the same putative compound was detected in both ionization modes, one representative feature was retained according to annotation evidence and QC reproducibility. Differential-feature analysis and downstream enrichment were interpreted primarily using Benjamini–Hochberg false-discovery-rate-adjusted *q* values (30). Volcano plots were used for visualization only, with display thresholds of |log2FC| ≥ 1.5 and nominal *p* < 0.01. Putative LC–MS feature annotation was based on accurate MS^1^ mass, retention behavior, and the available MS^2^ annotation evidence from in-house and public libraries. Annotation confidence was reported according to the Schymanski framework (31). No Level-1 identifications were claimed because authentic reference standards were not analyzed for all reported compounds under identical chromatographic and mass-spectrometric conditions. For the 15 detected LC–MS features, L01–L14 were conservatively treated as Level-3 tentative candidates, whereas L15 remained unassigned because the supplied annotation export contained a mass/formula inconsistency. These feature codes, rather than unverified compound identities, were used in the association analysis. Ionization mode, retention time, observed m/z, formula, QC reproducibility, confidence level, and verification notes are provided in Supplementary Table S7.

### 2.6 Broadly targeted GC–MS volatile profiling

Tea samples stored at −80 °C were ground under liquid nitrogen, and approximately 500 mg of powdered material was transferred to a 20 mL headspace vial containing 2 mL of saturated NaCl solution and 20 μL of 2-octanol internal-standard solution (10 μg/mL). Volatile compounds were enriched by automated headspace solid-phase microextraction before GC–MS analysis, consistent with established tea volatile-profiling workflows (32).

Samples were first equilibrated at 60 °C for 5 min, then extracted for 15 min at 60 °C using a 120 μm DVB/CAR/PDMS SPME Arrow fiber, and finally desorbed in the injector at 250 °C for 5 min. Fibers were conditioned at 250 °C before use, and newly installed fibers were preconditioned for 2 h.

Gas chromatographic separation was performed on a DB-5MS capillary column (30 m × 0.25 mm × 0.25 μm; Agilent J&W Scientific, Folsom, CA, USA) using helium as the carrier gas at 1.2 mL/min. The injector temperature was maintained at 250 °C, and the solvent delay was set at 3.5 min. The oven program was as follows: initial holding at 40 °C for 3.5 min; heating at 10 °C/min to 100 °C; then at 7 °C/min to 180 °C; followed by 25 °C/min to 280 °C; and holding at 280 °C for 5 min.

Mass spectrometric detection was conducted in electron ionization mode (70 eV), with ion-source, quadrupole, and transfer-line temperatures set to 230, 150, and 280 °C, respectively. Study samples were analyzed using a broadly targeted selected-ion-monitoring strategy based on a predefined in-house volatile-target database. For each target, one quantifier ion and the available qualifier ion(s) were monitored within predefined acquisition segments based on characteristic electron-ionization fragmentation patterns. The designated quantifier ion was used for peak integration, and peak areas were normalized to the 2-octanol internal standard for relative semi-quantification. Putative target assignments were based on the in-house target list and the available retention-index and target-ion criteria. Study samples were acquired exclusively in SIM mode, and no additional full-scan GC–MS dataset was collected; consequently, full-spectrum NIST library searching was not performed for individual study samples and no sample-specific NIST matching scores were generated. No compound-specific external calibration curves or analyte-specific response factors were applied; therefore, the reported GC–MS values represent internal-standard-corrected relative semi-quantitative abundances rather than absolute concentrations. The results are described as broadly targeted GC–MS/SIM volatile profiles within the predefined target range rather than comprehensive untargeted volatilomics. Supplementary Table S8 reports the available retention-index, quantifier-ion, qualifier-ion, and QC metadata. Chromatographic retention times and additional qualifier ions not present in the supplied target export were not estimated.

### 2.7 PCR amplification, sequencing, and bioinformatics

Bacterial 16S rRNA gene fragments were amplified using the primer pair F: AACMGGATTAGATACCCKG and R: ACGTCATCCCCACCTTCC, whereas fungal ITS regions were amplified using F: CTT-GGTCATTTAGAGGAAGTAA and R: GCTGCGTTCTTCATCGATGC.

PCR amplification was performed in a 25 μL reaction system containing Q5 High-Fidelity DNA Polymerase, 5 × reaction buffer, 5 × High GC buffer, dNTPs, template DNA, primers, and nuclease-free water. For bacteria, the thermocycling program consisted of an initial denaturation at 98 °C for 5 min, followed by 25 cycles of 98 °C for 30 s, 52 °C for 30 s, and 72 °C for 45 s, with a final extension at 72 °C for 5 min. For fungi, amplification started with denaturation at 98 °C for 5 min, followed by 30 cycles of 98 °C for 30 s, 55 °C for 45 s, and 72 °C for 45 s, followed by a final extension at 72 °C for 5 min. Amplicons were checked by 2% agarose gel electrophoresis, purified using an Axygen Gel Extraction Kit, and sequenced on the Illumina MiSeq platform.

Raw reads were processed using QIIME 2 (v2024.2) (33). Demultiplexed sequences were quality filtered, denoised, merged, and screened for chimeras using DADA2 to generate amplicon sequence variants (ASVs) (34). Taxonomic annotation was conducted using SILVA r138.1 for bacteria and UNITE v9 for fungi (35, 36). ASV tables were rarefied to an even sequencing depth before alpha- and beta-diversity analyses. Alpha-diversity indices were calculated in QIIME 2, whereas beta diversity was estimated using Bray–Curtis dissimilarity and visualized by principal coordinates analysis. Group differences were evaluated by PERMANOVA with 9,999 permutations (37, 38). Bacterial functional potential was inferred using PICRUSt2 at the COG and KEGG levels and was interpreted as predicted rather than directly measured function (39).

### 2.8 Statistics, sensory evaluation, and multi-omics integration

To complement the instrumental analyses, sensory evaluation was conducted on 24 November 2025 following established sensory–metabolomics applications in black tea and in accordance with the Chinese national standard GB/T 23776-2018 (40, 41). The panel consisted of six assessors with formal training and practical experience in black-tea quality evaluation. One assessor held a Level-1 Tea Appraiser qualification, whereas the other five assessors had completed formal training in tea sensory evaluation. All assessors were familiar with the procedures and scoring criteria specified in GB/T 23776-2018. Sample identities were concealed using randomized three-digit codes assigned by a researcher who retained the code key but did not participate in infusion preparation, sample presentation, or sensory scoring. Tea infusions were prepared and presented by a separate staff member who was unaware of cultivar identity. Cultivar identity and other sample information were not disclosed to the assessors, and the code key was not released until all sensory scores had been recorded. Samples were presented in randomized order, and each assessor completed the evaluation independently without discussion. Panel qualifications and the blinding procedure are summarized in Supplementary Table S5.

Appearance, liquor color, aroma, taste, and infused-leaf quality were scored using the attribute-specific criteria specified in GB/T 23776-2018. The weighted overall score was calculated as 25% appearance, 10% liquor color, 25% aroma, 30% taste, and 10% infused-leaf quality. The corresponding score ranges, operational quality descriptors, and weights, including the criteria distinguishing the 90–99 and 80–89 aroma-score ranges, are provided in Supplementary Table S4.

Profile-level inter-rater reliability was assessed across the 10 sample–attribute combinations, comprising two fermented samples and five primary sensory attributes, using a two-way random-effects, absolute-agreement intraclass correlation coefficient. Both the average-measures coefficient for the six-member panel, ICC(2,6), and the corresponding single-measure coefficient, ICC(2,1), were calculated. Differences between G1F and G2F were evaluated using paired-samples *t* tests. Effect sizes were expressed as Cohen’s *dz*., calculated as the mean assessor-level paired difference divided by the standard deviation of the paired differences. Leave-one-assessor-out analyses were conducted to evaluate whether the results were disproportionately influenced by any individual assessor, and Wilcoxon signed-rank tests were performed as non-parametric sensitivity analyses. Reliability estimates and sensitivity results are provided in Supplementary Table S6.

For cross-omics integration, named genera eligible for association testing were predefined using a prevalence threshold of ≥50% of samples and a mean relative abundance threshold of ≥0.1%. Chloroplast, mitochondrial, and other non-target bacterial entries were excluded. Multiplicative zero replacement was applied to the full retained genus-level composition, followed by centered log-ratio transformation. The log-ratio transformation was used because conventional correlations applied directly to microbial relative-abundance data may generate compositional artifacts (42–44). LC–MS and GC–MS variables were log2(x + 1)-transformed. To limit the multiple-testing burden, the analysis was restricted to 15 QC-qualified, flavor-associated features from each analytical platform. These features were predefined before association testing according to QC reproducibility, annotation confidence, and documented relevance to tea aroma, taste, or color chemistry. No feature was selected on the basis of its microbiota–metabolite correlation coefficient or associated *p* value. For each genus and metabolite feature, transformed values across the 12 matched samples were separately residualized using the model value ~ Cultivar + Stage, and Pearson correlations were calculated between the corresponding residuals. The resulting coefficients are referred to as adjusted partial correlations. *p* values were corrected using the Benjamini–Hochberg procedure separately within the four analysis families: bacteria–LC–MS, bacteria–GC–MS, fungi–LC–MS, and fungi–GC–MS. Associations meeting both |adjusted partial r| ≥ 0.40 and BH-FDR *q* < 0.05 were considered significant. Complete results for all tested genus–feature pairs are provided in Supplementary Table S9. For the within-group bacterial co-occurrence diagrams, Spearman correlations were calculated among genera separately in each group, and edges satisfying |*ρ*| ≥ 0.6 and BH-FDR *q* < 0.05 were displayed. Because each group contained only three biological replicates, these networks were retained solely as exploratory descriptive visualizations; they were not used for formal network comparison, ecological-interaction inference, or mechanistic conclusions. Unless otherwise specified, statistical significance was defined as *q* < 0.05.

## 3 Results

### 3.1 Sequence statistics and diversity patterns of bacterial and fungal communities

**Figure 1:**
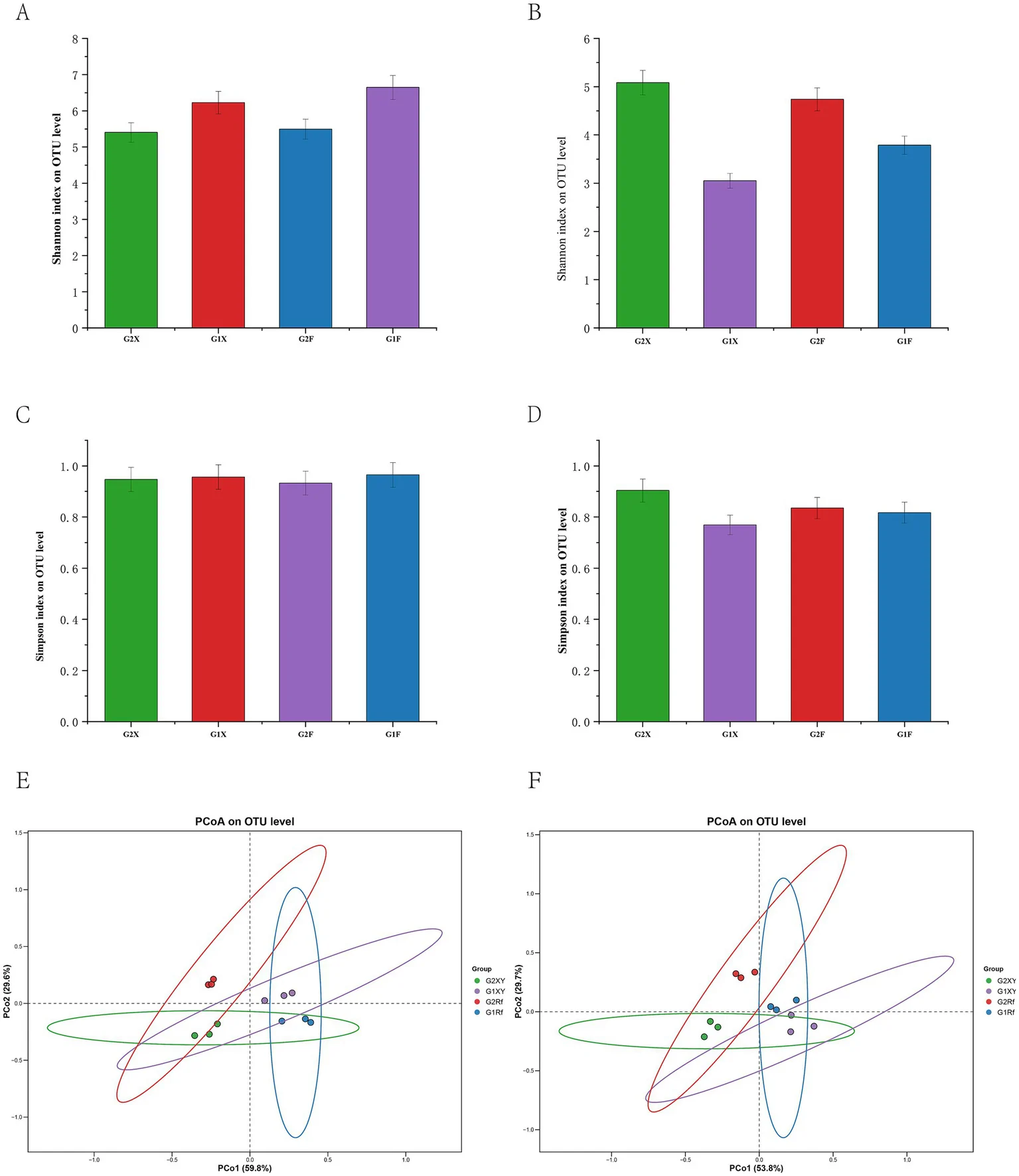
Alpha and beta diversity of surface-sterilized leaf-associated microbiota in Gougunao tea. **(A)** Bacterial Shannon index. **(B)** Fungal Shannon index. **(C)** Bacterial Simpson index. **(D)** Fungal Simpson index. **(E)** Principal coordinates analysis (PCoA) of bacterial communities based on Bray–Curtis dissimilarity. **(F)** Principal coordinates analysis (PCoA) of fungal communities based on Bray–Curtis dissimilarity.

Amplicon sequencing was used to characterize bacterial and fungal communities associated with fresh (X) and fermented (F) leaves from the two cultivars, G1 and G2. In the bacterial dataset, 984,569 high-quality 16S rRNA gene reads were obtained, with an average of approximately 82,047 reads per sample, yielding 3,525 bacterial ASVs. Taxonomic annotation assigned these sequences to 5 phyla, 18 classes, 42 orders, 96 families, and 210 genera. Across all treatment groups, Sphingomonas was the dominant genus, suggesting that differences between fresh and fermented leaves more likely reflected shifts in the relative structure of a pre-existing leaf-associated community rather than complete replacement by unrelated taxa. Alpha-diversity indices and PCoA ordinations are shown in Figures 1A–F.

For the fungal dataset, ITS sequencing of the same 12 samples generated 1,011,078 valid reads, with a mean of 84,256 reads per sample, resulting in 913 fungal ASVs. These sequences were assigned to 2 phyla, 10 classes, 28 orders, 61 families, and 85 genera. Compared with fungi, bacterial communities showed greater genus-level richness, which was also reflected by their broader dispersion in *β*-diversity space.

PERMANOVA based on Bray–Curtis distance matrices demonstrated strong overall separation among the four groups (G1X, G2X, G1F, and G2F) for both bacteria and fungi. For bacteria, the overall group effect was highly significant (pseudo-*F* = 216.68, *R*^2^ = 0.9878, *p* = 0.0002). Bacterial community composition differed significantly between G1 and G2 (pseudo-*F* = 13.63, *R*^2^ = 0.5768, *p* = 0.0025), whereas stage did not reach significance in the distance-based model (pseudo-*F* = 2.07, *R*^2^ = 0.1718, *p* = 0.1281). For fungi, the overall group effect was likewise significant (pseudo-*F* = 227.29, *R*^2^ = 0.9884, *p* = 0.0002), and fungal community composition differed significantly according to both cultivar (pseudo-*F* = 9.28, *R*^2^ = 0.4813, *p* = 0.0025) and stage (pseudo-*F* = 3.74, *R*^2^ = 0.2721, *p* = 0.0231). Taken together, significant differences between the two tested cultivars were detected in both microbial kingdoms, whereas stage-related variation was more clearly captured in fungi than in bacteria.

### 3.2 Bacterial community composition across fresh and fermented leaves

**Figure 2:**
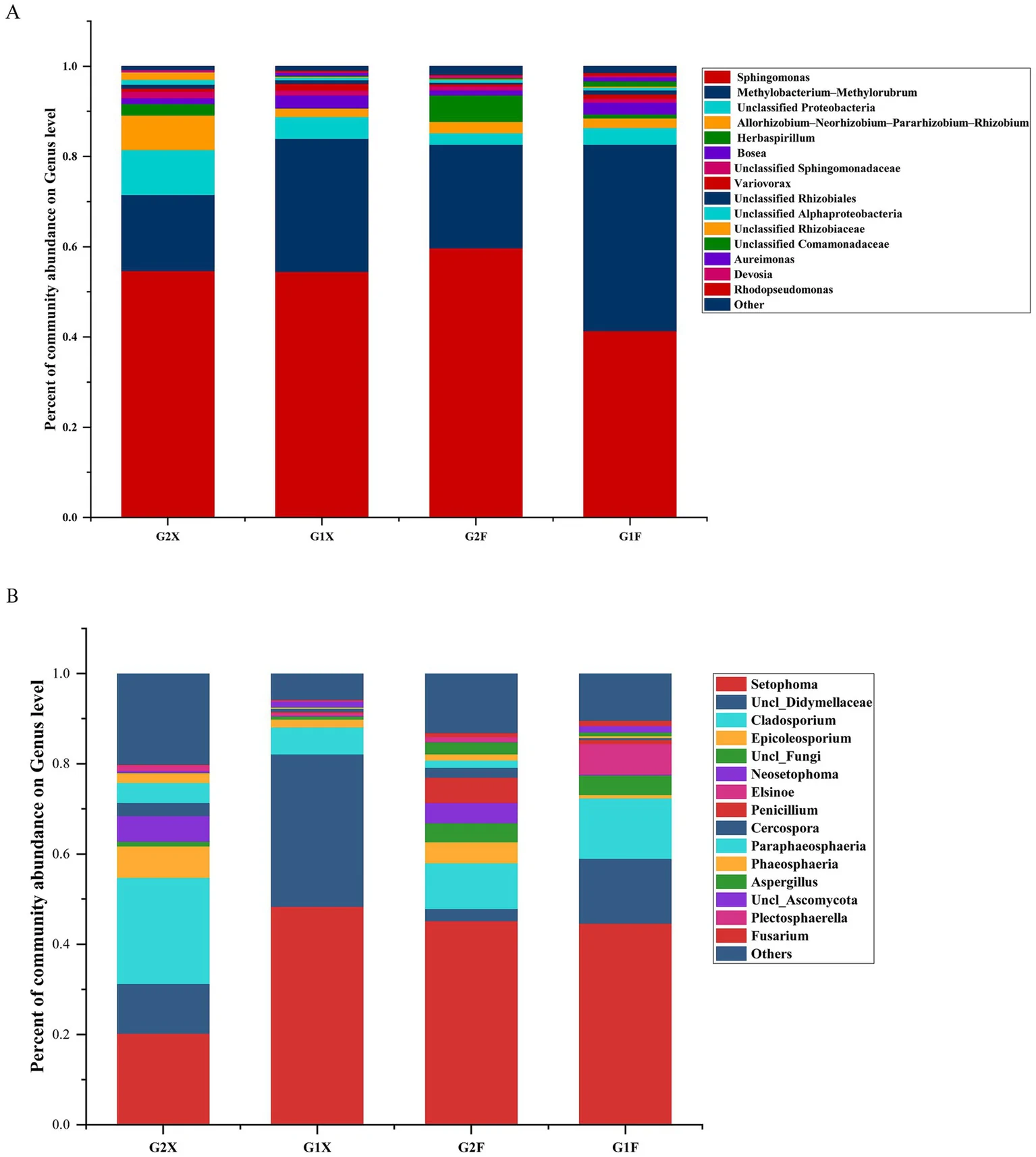
Genus-level composition of surface-sterilized leaf-associated microbiota in tea samples. **(A)** Bacterial communities. **(B)** Fungal communities. G1X and G2X denote fresh leaves of cultivars G1 and G2, respectively, whereas G1F and G2F denote the corresponding fermented leaves after black tea processing.

The genus-level bacterial profiles further supported the separation patterns indicated by PCoA and PERMANOVA. A pronounced overall group effect and a significant cultivar effect were detected, whereas stage alone was not significant in the distance-based model. At the genus level, based on the top 20 taxa shown in Figure 2A, several core groups, including Sphingomonas, Methylobacterium, Allorhizobium–Rhizobium, and Herbaspirillum, were present in all treatments, although their relative abundances varied according to cultivar and processing stage.

**Figure 3:**
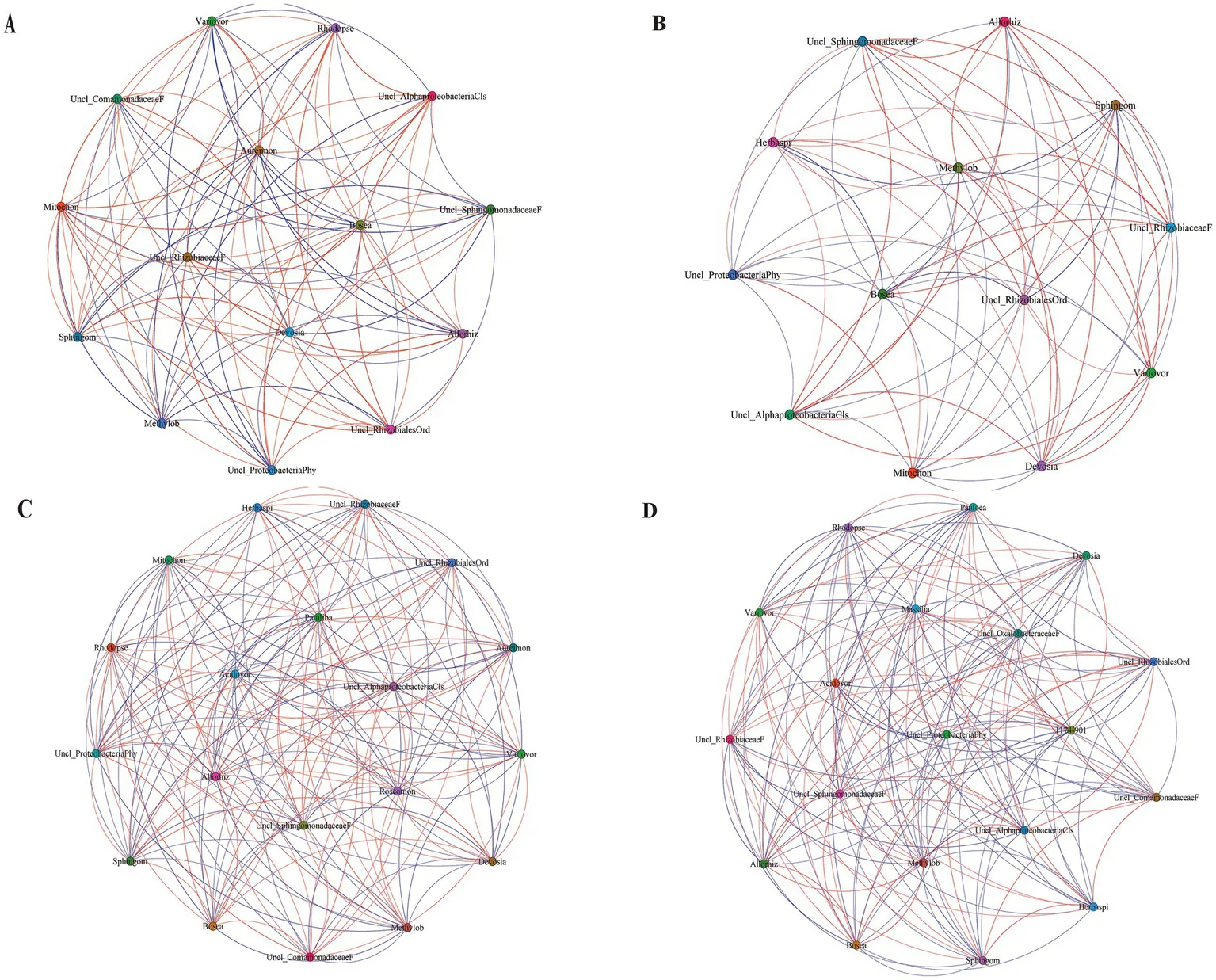
Exploratory genus-level bacterial co-occurrence diagrams with phylum-level node annotation **(A)** G1X; **(B)** G2X; **(C)** G1F; **(D)** G2F. Spearman correlations were calculated among genera within each group, and displayed edges met |*ρ*| ≥ 0.6 and BH-FDR *q* < 0.05. Red and blue edges indicate positive and negative correlations, respectively. Nodes represent genera, including unclassified lineages, and node colours denote bacterial phyla. Because each group contained only three biological replicates, these diagrams are presented for descriptive visualization only and should not be interpreted as statistically robust networks, direct ecological interactions, or evidence of group-level differences in network topology. PERMANOVA statistics for bacterial and fungal communities are provided in Supplementary Tables S1, S2.

More specifically, Sphingomonas and Rhizobium-related taxa accounted for a larger share of the fermented-leaf communities, whereas Methylobacterium contributed more prominently to the fresh-leaf samples. The within-group co-occurrence diagrams are retained in Figure 3 as exploratory descriptive visualizations of the correlation patterns observed in these samples. Because each diagram was based on only three biological replicates, differences in network density, connectivity, or organization were not statistically compared and are not interpreted as robust ecological interactions. The principal bacterial-community conclusions are therefore based on taxonomic composition, ordination, and PERMANOVA rather than on the network diagrams.

### 3.3 Fungal community composition across fresh and fermented leaves

Fungal relative abundance was expressed as the proportion of total sequencing reads. At the genus level (Figure 2B), fungal communities were dominated by a smaller set of taxa than bacterial communities and showed more evident stage-associated shifts. In fresh leaves, G2X displayed a relatively balanced composition, with Cladosporium, Setophoma, unclassified Didymellaceae, and Epicoleosporium all representing major contributors. By contrast, G1X was largely dominated by Setophoma and unclassified Didymellaceae.

After black tea processing, Setophoma remained the major genus in both fermented-leaf groups, although the secondary taxa differed between cultivars. In G2F, the relative abundance of unclassified Didymellaceae decreased, whereas Penicillium and Aspergillus increased. In G1F, unclassified Didymellaceae also declined, while Cladosporium and Elsinoe became more abundant. In both cultivars, the proportion of unclassified fungal reads increased slightly following processing. A global fungal co-occurrence diagram annotated at the phylum level is presented in Supplementary Figure S1 only as an exploratory overview; given the limited replication, it is not used to infer stable ecological interactions.

### 3.4 Predicted functional profiles and metabolite pathway context

**Figure 4:**
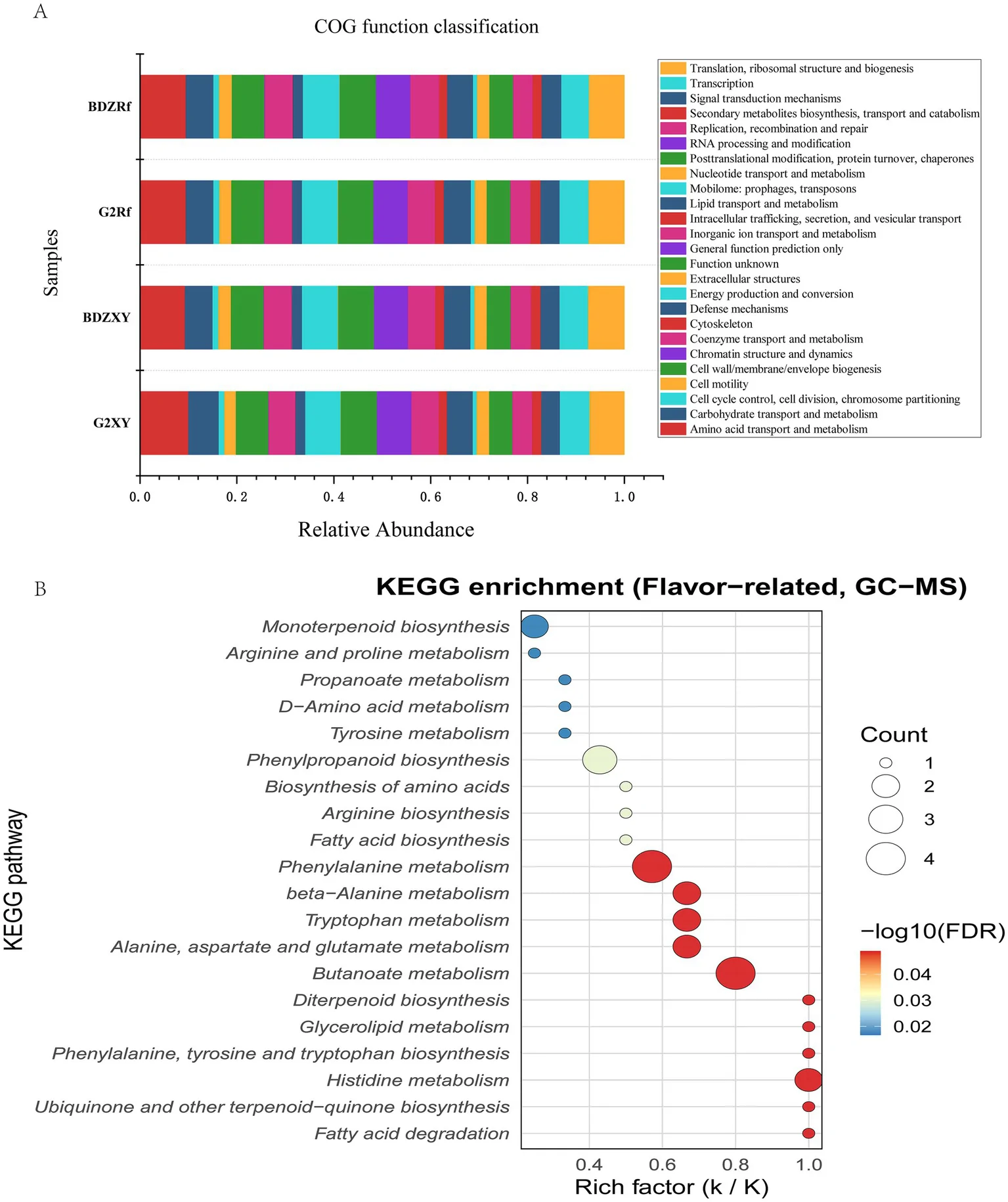
Predicted bacterial functional profiles and metabolite pathway context across cultivars and stages. **(A)** COG functional categories inferred from 16S rRNA gene data using PICRUSt2 and shown as mean predicted relative abundance for G2X, G1X, G2F, and G1F. PICRUSt2 values represent predicted functional potential inferred from 16S rRNA gene composition and should not be interpreted as directly measured microbial activity. **(B)** KEGG level-3 enrichment of flavor-associated chemical features. The x-axis indicates the rich factor, dot size represents the number of mapped features, and color indicates −log10 (BH-FDR-adjusted *q*). Only significantly enriched categories are shown. The KEGG enrichment results provide broad biochemical context and are not used as evidence that the corresponding metabolic reactions were performed by the detected microorganisms.

PICRUSt2-based inference produced broadly comparable distributions of predicted functional categories across the four cultivar–stage groups at the coarse COG level (Figure 4A). Major predicted categories included energy production and conversion, amino acid transport and metabolism, carbohydrate transport and metabolism, nucleotide metabolism, lipid metabolism, cofactor metabolism, and cellular defense. Because these profiles were inferred from 16S rRNA gene composition rather than directly measured by metagenomics, metatranscriptomics, or enzyme assays, they were treated as prediction-based context rather than as a principal biological finding.

Metabolite-centered KEGG enrichment identified broad level-3 categories related mainly to amino acid, carbohydrate, terpenoid, and phenylpropanoid metabolism (Figure 4B). These categories provided general biochemical context for the detected features but were not interpreted as evidence that the corresponding reactions were performed by the detected microorganisms. More specific biological information was obtained from the compound-level cultivar contrasts and the adjusted genus–feature association patterns described in Sections 3.5–3.9.

### 3.5 LC–MS metabolome: cultivar contrasts at fresh and fermented stages

**Figure 5:**
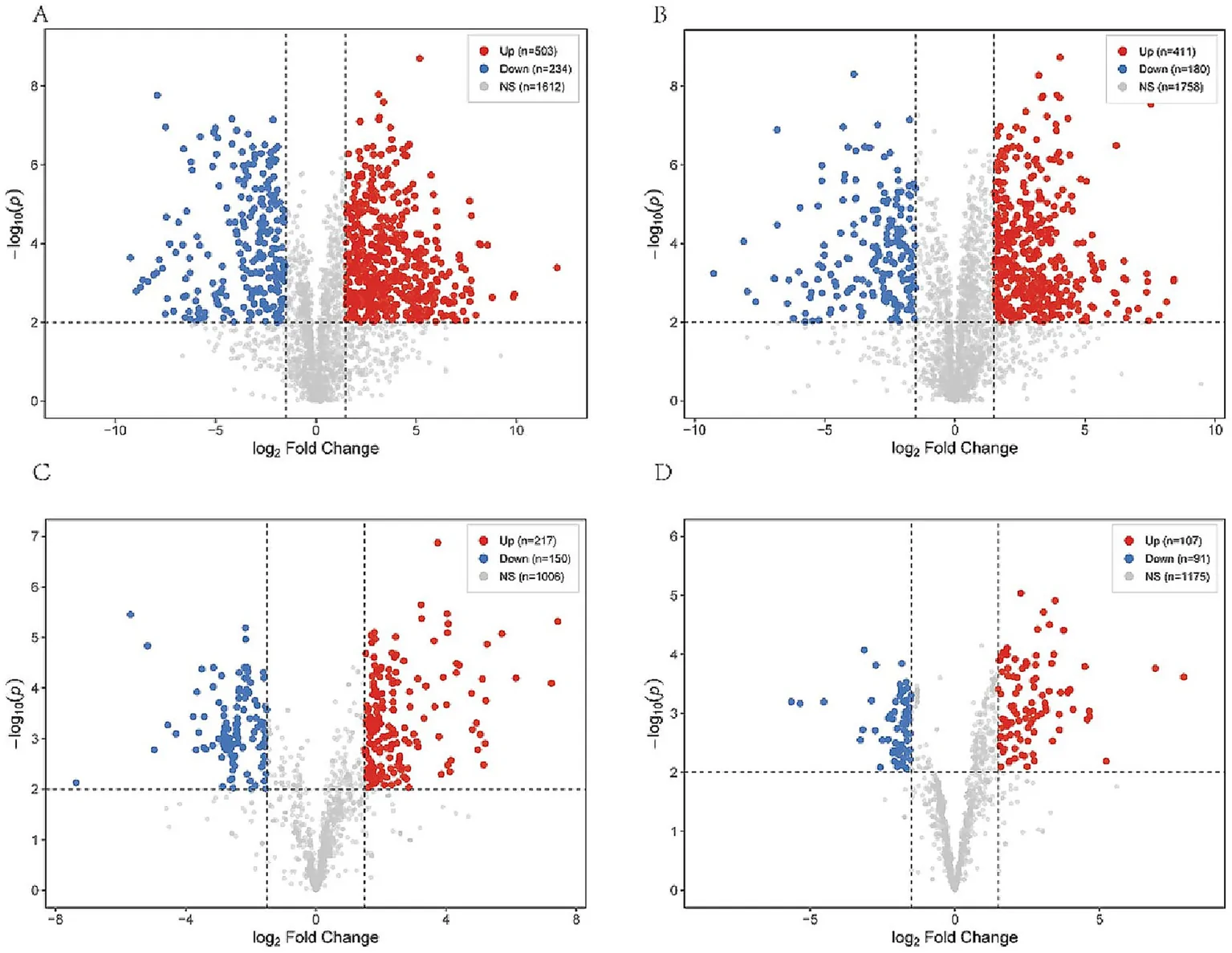
Volcano plots of cultivar contrasts across analytical platforms. (A, B) untargeted LC–MS metabolomics: **(A)** G2X vs. G1X; **(B)** G2F vs. G1F. (C, D) broadly targeted GC–MS volatile profiling in SIM mode: **(C)** G2X vs. G1X; **(D)** G2F vs. G1F. Vertical dashed lines indicate the fold-change threshold (|log_2_*FC*| = 1.5), and the horizontal dashed line indicates the nominal significance threshold (*p* = 0.01). Features passing both display thresholds are highlighted, and representative putatively annotated features are labelled.

Untargeted LC–MS features were compared between cultivars at both the fresh- and fermented-leaf stages using volcano plots (Figures 5A,B). For visualization, features meeting the criteria of |log2FC| ≥ 1.5 and nominal *p* < 0.01 were highlighted. However, formal differential-feature analysis and downstream enrichment interpretation were based on BH-FDR-adjusted *q* values unless otherwise indicated. In the fresh-leaf comparison (G2X vs. G1X; Figure 5A), numerous features differed between the two cultivars, indicating that their metabolite backgrounds were already distinct before black tea processing. In the fermented-leaf comparison (G2F vs. G1F; Figure 5B), cultivar-associated differences remained detectable under the same manufacturing conditions. Representative putatively annotated features with available annotation evidence were labeled to illustrate the principal chemical categories underlying the observed separation; these labels were not treated as Level-1 confirmations.

At the fresh-leaf stage, G2X exhibited relatively higher levels of amino acid- and organic acid-related features, whereas G1X showed comparatively stronger signals for galloylated catechin- and phenolic-associated compounds. In addition, several putative glycosidic features, inferred from retention behavior and characteristic neutral-loss patterns in MS^2^ spectra, appeared more prominent in G2X. In fermented leaves, the differential feature space shifted toward metabolites more closely associated with aroma and color formation. G2F displayed relatively higher levels of monoterpene- and short-chain fatty acid-related features, whereas G1F showed stronger signals for polyphenol oxidation-related compounds together with selected glycosidic or heterocyclic features. Overall, the LC–MS results indicate that cultivar-associated chemical divergence was already present in fresh leaves and remained evident after fermentation, with the dominant contrast in fermented samples moving toward aroma- and color-associated branches.

### 3.6 Broadly targeted GC–MS/SIM volatile profiles across fresh and fermented leaves

Broadly targeted GC–MS/SIM analysis was used to compare predefined volatile profiles between cultivars at the fresh- and fermented-leaf stages (Figures 5C,D). To maintain consistency with LC–MS presentation, volcano plots highlighted volatile features exceeding |log2FC| ≥ 1.5 and nominal *p* < 0.01, whereas formal downstream interpretation relied primarily on BH-FDR-adjusted *q* values unless otherwise specified. In fresh leaves (G2X vs. G1X; Figure 5C), a subset of volatile compounds already differed between cultivars. In fermented leaves (G2F vs. G1F; Figure 5D), additional cultivar-associated differences were observed, indicating that cultivar-specific volatile signatures persisted after black tea processing.

In the fresh-leaf comparison, G2X showed relatively stronger signals for subsets of amino acid- and lipid-related volatiles, whereas G1X was comparatively enriched in selected phenylpropanoid- and terpenoid-related volatile compounds. After processing, the volatile contrast shifted toward aroma-related chemical branches. G2F exhibited relatively higher abundances of monoterpene-related alcohols and esters together with selected short-chain fatty acid derivatives, whereas G1F showed relatively stronger signals for certain oxidation- and Maillard-associated volatiles. Collectively, the GC–MS data support differences between the two tested cultivars at both stages and indicate that the composition of discriminatory volatile features changes during processing.

### 3.7 Sensory evaluation of fermented samples

Profile-level inter-rater reliability across the 10 sample–attribute combinations was ICC(2,6) = 0.870 for the mean ratings of the six assessors, whereas the corresponding single-measure coefficient was ICC(2,1) = 0.526. At the panel level, G2F received higher mean scores than G1F for all five primary sensory attributes and for the weighted overall score (Table 1). The largest difference among the primary sensory attributes was observed for aroma. G2F scored 93.50 ± 1.05, compared with 89.67 ± 1.03 for G1F, corresponding to a mean paired difference of 3.83 points (95% CI, 3.04–4.62; *p* < 0.001; Cohen’s *dz*. = 5.09). Taste showed a mean paired difference of 2.83 points (95% CI, 1.80–3.87; *p* = 0.001; Cohen’s *dz*. = 2.88). Differences were also observed for appearance, liquor color, and infused-leaf quality, with Cohen’s *dz*. values of 3.23, 1.52, and 1.52, respectively. The weighted overall score was 91.48 ± 0.67 for G2F and 88.73 ± 0.64 for G1F, corresponding to a mean paired difference of 2.74 points (95% CI, 2.43–3.06; *p* < 0.001; Cohen’s *dz* = 9.17). In the leave-one-assessor-out analyses, all mean differences remained positive and all paired comparisons retained nominal significance after exclusion of any single assessor. Wilcoxon sensitivity analyses supported the differences in appearance, liquor color, aroma and taste, whereas the infused-leaf difference remained directionally consistent but did not reach significance (*p* = 0.063). These results provide panel-level sensory support for differences between the two fermented products, although the limited panel size warrants cautious interpretation.

**Table 1:** Sensory evaluation of black tea samples G1F and G2F based on a weighted scoring system (mean ± SD, *n* = 6).

Attribute	Weight (%)	G1F	G2F	Paired difference (95% CI)	*p* value	Cohen’s *d*z
Appearance	25	88.00 ± 1.41	89.67 ± 1.75	1.67 (1.12–2.21)	0.001	3.23
Liquor color	10	89.00 ± 2.97	91.67 ± 2.58	2.67 (0.83–4.50)	0.014	1.52
Aroma	25	89.67 ± 1.03	93.50 ± 1.05	3.83 (3.04–4.62)	<0.001	5.09
Taste	30	88.83 ± 0.75	91.67 ± 1.21	2.83 (1.80–3.87)	0.001	2.88
Infused leaf	10	87.67 ± 1.03	90.17 ± 0.75	2.50 (0.78–4.22)	0.014	1.52
Weighted overall score	–	88.73 ± 0.64	91.48 ± 0.67	2.74 (2.43–3.06)	<0.001	9.17

Values are expressed as assessor-level mean ± standard deviation (*n* = 6). Paired differences were calculated as G2F minus G1F. *p* values were obtained from paired-samples t tests, and Cohen’s *dz* was calculated as the mean paired difference divided by the standard deviation of the paired differences.

### 3.8 Adjusted bacterial genus–metabolite associations across LC–MS and GC–MS

**Figure 6:**
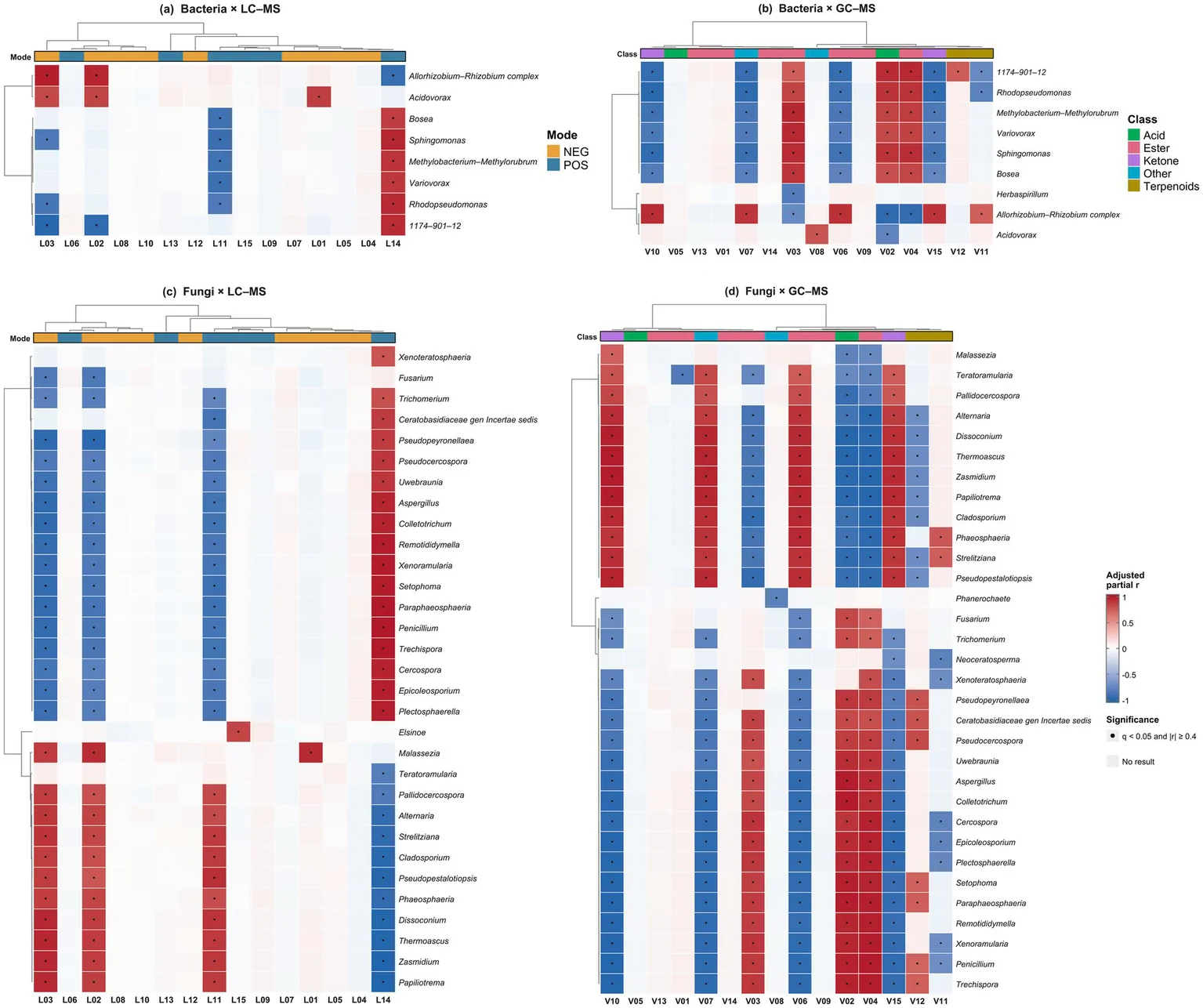
Adjusted associations between microbial genera recovered from surface-sterilized leaves and flavor-associated features in fresh and fermented leaves of Gougunao black tea: **(a)** Bacterial genera versus LC–MS features; **(b)** Bacterial genera versus broadly targeted GC–MS/SIM volatile features; **(c)** Fungal genera versus LC–MS features; **(d)** Fungal genera versus broadly targeted GC–MS/SIM volatile features. Associations were estimated using Pearson correlations between residuals after adjustment for cultivar and processing stage. Cell color indicates the adjusted partial correlation coefficient (*r*), with red and blue representing positive and negative associations, respectively. Black dots mark associations meeting both |adjusted partial *r*| ≥ 0.40 and Benjamini–Hochberg-adjusted *q* < 0.05. Pale cells represent non-significant associations, whereas light-grey cells indicate unavailable genus–feature pairs. LC–MS and GC–MS/SIM columns were clustered separately, and the same column order was retained across the bacterial and fungal panels for each analytical platform. L01–L15 denote the selected LC–MS features, whereas V01–V15 denote the selected QC-qualified volatile targets measured using the broadly targeted GC–MS/SIM workflow. Putative assignments, confidence levels, target-ion metadata, and verification notes are provided in Supplementary Tables S7, S8. Only genera with at least one significant adjusted association are shown; complete results for all tested genus–feature pairs are provided in Supplementary Table S9. Hierarchical clustering was used only to organize similar adjusted association profiles and should not be interpreted as evidence of direct functional interaction or causal network structure.

After compositional transformation and adjustment for cultivar and processing stage, the bacterial analysis identified a limited set of adjusted genus–feature associations across the two analytical platforms (Figures 6a,b). In the LC–MS panel, L14 showed positive associations with Bosea, Sphingomonas, Methylobacterium–Methylorubrum, Variovorax, Rhodopseudomonas, and 1174–901–12, but a negative association with the Allorhizobium–Rhizobium complex. L11 showed negative associations with several genera belonging to the former group, whereas L03 and L02 differed in direction between the Allorhizobium–Rhizobium complex or Acidovorax and the Sphingomonas- or Rhodopseudomonas-associated genera.

In the GC–MS panel, 1174–901–12, Rhodopseudomonas, Methylobacterium–Methylorubrum, Variovorax, Sphingomonas, and Bosea showed similar directional associations, including positive associations with V03, V02, and V04 and negative associations with V10, V07, V06, and V15. The Allorhizobium–Rhizobium complex showed the opposite direction for several of these volatile features. Thus, after adjustment for cultivar and processing stage, the displayed bacterial associations were concentrated in a limited number of opposing directional patterns. These associations remain exploratory and should not be interpreted as direct microbial effects.

### 3.9 Adjusted fungal genus–feature associations across LC–MS and GC–MS

Adjusted fungal associations showed two broadly opposing directional patterns across the LC–MS and GC–MS datasets (Figures 6c,d). In the LC–MS panel, Setophoma, Aspergillus, Penicillium, Pseudocercospora, and Colletotrichum showed mainly negative associations with L03, L02, and L11 but positive associations with L14. By contrast, Cladosporium, Alternaria, Phaeosphaeria, Dissoconium, Zasmidium, and Papiliotrema showed positive associations with L03, L02, and L11 and negative associations with L14.

A broadly similar directional contrast was observed in the GC–MS panel. Malassezia, Teratoramularia, Pallidocercospora, Alternaria, Dissoconium, Thermoascus, Zasmidium, Papiliotrema, and Cladosporium were generally positively associated with V10, V07, V06, and V15 and negatively associated with V02 and V04. In contrast, Setophoma, Paraphaeosphaeria, Penicillium, Trechispora, Aspergillus, and Colletotrichum showed the inverse direction for several features. These opposing patterns remained visible after adjustment for cultivar and processing stage, but they are presented as candidate genus–feature associations rather than reproducible ecological modules or evidence of direct microbial effects on flavor formation.

The displayed fungal associations were not reducible to a simple class-level contrast. Several Dothideomycetes-affiliated genera, including Cladosporium, Alternaria, Phaeosphaeria, and Dissoconium, showed one directional pattern, whereas Setophoma, which is also affiliated with Dothideomycetes, showed an opposing pattern together with the Eurotiomycetes-affiliated genera Aspergillus and Penicillium. Thus, the observed result was not a uniform Dothideomycetes–Eurotiomycetes dichotomy, but taxon-specific variation within and across fungal classes. This descriptive genus-level heterogeneity was not apparent from the broadly comparable PICRUSt2 functional-category profiles.

## 4 Discussion

### 4.1 Differences between the two tested cultivars in surface-sterilized leaf-associated microbial communities

The present study identified differences between the two tested Gougunao tea cultivars in microbial communities recovered from surface-sterilized fresh and fermented leaves. Ordination analysis together with PERMANOVA showed distinct separation among the experimental groups for both bacterial and fungal communities. In the current dataset, significant differences between G1 and G2 were detected in the structure of both microbial kingdoms, whereas a significant stage effect was detected only for fungi. These findings indicate that the two cultivars had different baseline microbial profiles and chemical precursor states under the conditions examined, and that processing-related changes were imposed on, rather than fully replacing, these initial differences. Because only two cultivars were included, these results should not be interpreted as a general cultivar-dependent pattern across Gougunao germplasm or black tea cultivars.

At the taxonomic level, bacterial communities in all groups were primarily dominated by genera such as Sphingomonas and Methylobacterium, whereas fungal assemblages exhibited more obvious redistribution across stages, especially among Dothideomycetes-associated genera including Setophoma and Cladosporium. This interkingdom difference is important for interpretation. In the present dataset, fungal communities appeared more responsive to processing stage at the distance-matrix level, whereas stage-related variation in bacteria was reflected more clearly in relative-abundance profiles than in the overall PERMANOVA main effect. Accordingly, bacterial stage-associated patterns should be interpreted conservatively and evaluated together with taxonomic composition and the adjusted cross-omics association results rather than being inferred solely from the distance-based model.

### 4.2 Cultivar-related divergence in metabolite profiles and sensory traits

The chemical results likewise indicated that divergence between G1 and G2 was already present before processing and remained detectable after fermentation. Both LC–MS and broadly targeted GC–MS/SIM analyses showed that the two cultivars differed in baseline metabolite composition at the fresh-leaf stage, including amino acid-, phenolic-, and terpenoid-associated branches. After black tea manufacture, these initial differences were still evident, but the discriminatory chemical features became more strongly represented in aroma- and color-related space. This pattern is consistent with the general understanding that black tea flavor formation is driven principally by endogenous enzymatic oxidation and aroma-precursor conversion (1, 2, 10, 11). The initial chemical composition of the leaves, which is influenced by genotype, environmental conditions, harvest season, and postharvest treatment, constrains the range and direction of subsequent transformations (12, 22, 24).

The sensory results were directionally consistent with the instrumental profiles. G2F received higher panel means for all five primary attributes and for the weighted overall score, with the largest difference observed for aroma, followed by taste. The six-assessor mean ratings showed good profile-level reliability, while the lower single-measure coefficient indicates that individual assessor scores were less stable than the panel average. Leave-one-assessor-out and non-parametric sensitivity analyses supported the overall direction of the cultivar contrast. Although the panel included one Level-1 Tea Appraiser and five formally trained assessors, the six-member local panel limits the broader generalizability of the sensory comparison. The sensory findings should therefore be interpreted as supportive panel-level evidence accompanying the instrumental analyses rather than as definitive evidence of a broadly generalizable cultivar effect.

### 4.3 Taxon-specific microbial–chemical associations beyond broad functional predictions

Previous studies have already documented microbial succession and microbial–quality relationships during black-tea processing (15–17). Integrated bacterial-community, volatile, correlation, and functional-prediction analyses have also been used to identify taxa potentially associated with black-tea aroma formation (18). Related microbiome–metabolome studies in Pu-erh and Fuzhuan brick teas have linked microbial succession with carbohydrate utilization and broader metabolite changes (20, 21). Accordingly, the contribution of the present study does not lie simply in combining microbiome and chemical datasets.

The specific contribution lies in the controlled comparison of two Gougunao tea cultivars subjected to the same manufacturing procedure and in testing whether microbial–chemical relationships remained detectable after accounting for microbiome compositionality, cultivar background, and processing stage. This approach separated broad group-level differences from a more restricted set of adjusted genus–feature covariation patterns.

The contrast between the PICRUSt2 and adjusted association results was informative. Although the bacterial communities showed broadly comparable predicted functional-category distributions at the coarse COG level, the genus-level association matrices contained differentiated and opposing directional patterns. Thus, coarse functional prediction did not capture the taxon-specific heterogeneity suggested by the adjusted cross-omics results.

The fungal results provided a clear example of this heterogeneity. Several Dothideomycetes-affiliated genera showed one directional association pattern, whereas Setophoma, also a member of Dothideomycetes, showed the opposing pattern together with Aspergillus and Penicillium. The principal observation was therefore not a general contrast between two fungal classes, but genus-specific variation within and across fungal classes. Given the limited sample size, these patterns should be regarded as exploratory and require validation in larger independent datasets.

Nevertheless, the adjusted associations should not be interpreted as evidence that the detected microorganisms directly produced or transformed the corresponding compounds. Similar microbiome–metabolome studies in fermented teas have used correlation analysis to identify candidate relationships and provide a basis for subsequent fermentation research (18, 20, 21). Residual associations in the present study may also reflect unmeasured host characteristics, local tissue conditions, or coordinated responses to processing.

### 4.4 Limitations and future perspectives

Several limitations should be considered. First, only two Gougunao tea cultivars were examined, each with three biological replicates per stage. The observed differences therefore apply to G1 and G2 under the present cultivation and processing conditions and do not establish a general cultivar-dependent pattern. Second, the experimental design included only fresh and fermented/processed samples and therefore did not resolve intermediate manufacturing stages. Third, the within-group co-occurrence diagrams were based on only three biological replicates per group. They were retained solely as exploratory descriptive visualizations and cannot support robust inference regarding ecological interactions or differences in network topology. Fourth, the operational definition of the leaf-associated microbiota was constrained by the surface-sterilization validation procedure. The absence of detectable microbial growth in the final rinse provided only a culture-based procedural check and could not exclude residual microbial DNA or determine taxon-specific removal efficiency. Surface-sterilization conditions may themselves influence the microbial profiles recovered from tea tissues (45). The detected communities should therefore be interpreted as surface-sterilized leaf-associated microbiota rather than strictly resident or endophytic communities. Fifth, bacterial functional interpretation relied on PICRUSt2 prediction rather than direct metagenomic, transcriptomic, enzymatic, or metabolic-activity measurements (39)^.^ Sixth, LC–MS compound identities were putatively annotated without complete authentic-standard confirmation; the Figure 6 LC–MS variables should therefore be interpreted primarily as feature codes, with confidence levels and verification notes reported in Supplementary Table S7. Seventh, the GC–MS study samples were acquired exclusively in SIM mode. The volatile analysis was limited to compounds included in the predefined target-ion database, no analyte-specific external calibration curves were applied, and the reported abundances are relative semi-quantitative values rather than absolute concentrations. Compounds outside the target range could not be retrospectively detected or evaluated through full-spectrum library searching. Eighth, the sensory comparison was based on a six-member local assessor panel. Although the assessors had formal qualifications or training and the reliability and sensitivity analyses supported the internal consistency of the results, the limited panel size and local assessor background restrict broader generalization. Finally, the cross-omics results represent adjusted association patterns rather than causal mechanisms. Although compositional transformation and adjustment for cultivar and processing stage reduced important analytical biases, residual associations may still reflect unmeasured host or processing-related factors. Previous studies have demonstrated that altered fermentation regimes, controlled microbial inoculation, and re-rolling can modify tea flavor and metabolic profiles (46–48); however, the present genus–feature relationships provide a basis for further investigation and do not establish direct microbial causality.

## 5 Conclusion

In summary, cultivar-associated differences in surface-sterilized leaf-associated microbial communities and flavor-related chemistry were already evident in fresh Gougunao tea leaves and remained detectable after a shared black tea manufacturing procedure. Significant cultivar-associated variation was detected in both bacterial and fungal community structures, whereas processing-stage-associated variation was more clearly expressed in fungal communities. LC–MS and broadly targeted GC–MS/SIM analyses further showed that cultivar-associated chemical differences persisted after processing, with the major contrasts in fermented leaves shifting toward aroma- and color-associated features.

Although PICRUSt2 indicated broadly comparable predicted bacterial functional-category profiles among groups, adjusted cross-omics analysis revealed reciprocal and taxon-specific genus–feature association patterns across the volatile and non-volatile datasets. In particular, genera belonging to the same fungal class occurred in opposing chemical-association modules, demonstrating genus-level heterogeneity that was not captured by coarse functional prediction. These findings provide new insight into microbial–chemical covariation in Gougunao black tea and establish a basis for further investigation of the corresponding genus–feature relationships. Direct microbial causality remains to be established.

## Data Availability

The data presented in this study can be found in the NCBI (https://www.ncbi.nlm.nih.gov), accession number PRJNA1506846.

## References

[1] Aaqil M Peng C Kamal A Nawaz T Zhang F Gong J. Tea harvesting and processing techniques and its effect on phytochemical profile and final quality of black tea: a review. Foods. (2023) 12:4467. doi: 10.3390/foods12244467, 38137271 PMC10743253

[2] Long P Rakariyatham K Ho C-T Zhang L. Thearubigins: formation, structure, health benefit and sensory property. Trends Food Sci Technol. (2023) 133:37–48. doi: 10.1016/j.tifs.2023.01.013

[3] Mozumder NHMR Lee J-E Hong Y-S. A comprehensive understanding of *Camellia sinensis* tea metabolome: from tea plants to processed teas. Annu Rev Food Sci Technol. (2025) 16:379–402. doi: 10.1146/annurev-food-111523-121252, 39874609

[4] Naveed M BiBi J Kamboh AA Suheryani I Kakar I Fazlani SA . Pharmacological values and therapeutic properties of black tea (*Camellia sinensis*): a comprehensive overview. Biomed Pharmacother. (2018) 100:521–31. doi: 10.1016/j.biopha.2018.02.048, 29482046

[5] Radeva-Ilieva M Stoeva S Hvarchanova N Georgiev KD. Green tea: current knowledge and issues. Foods. (2025) 14:745. doi: 10.3390/foods14050745, 40077449 PMC11899301

[6] Dunn WB Bailey NJC Johnson HE. Measuring the metabolome: current analytical technologies. Analyst. (2005) 130:606. doi: 10.1039/b418288j, 15852128

[7] Nicholson JK Lindon JC. Metabonomics. Nature. (2008) 455:1054–6. doi: 10.1038/4551054a, 18948945

[8] Lee J-E Lee B-J Chung J-O Hwang J-A Lee S-J Lee C-H . Geographical and climatic dependencies of green tea (*Camellia sinensis*) metabolites: a ^1^H NMR-based metabolomics study. J Agric Food Chem. (2010) 58:10582–9. doi: 10.1021/jf102415m, 20828156

[9] Liang Y Lu J Zhang L Wu S Wu Y. Estimation of black tea quality by analysis of chemical composition and colour difference of tea infusions. Food Chem. (2003) 80:283–90. doi: 10.1016/S0308-8146(02)00415-6

[10] Ho C-T Zheng X Li S. Tea aroma formation. Food Sci Human Wellness. (2015) 4:9–27. doi: 10.1016/j.fshw.2015.04.001

[11] Zeng L Watanabe N Yang Z. Understanding the biosyntheses and stress response mechanisms of aroma compounds in tea (*Camellia sinensis*) to safely and effectively improve tea aroma. Crit Rev Food Sci Nutr. (2019) 59:2321–34. doi: 10.1080/10408398.2018.1506907, 30277806

[12] Wei K Wang L Zhou J He W Zeng J Jiang Y . Catechin contents in tea (*Camellia sinensis*) as affected by cultivar and environment and their relation to chlorophyll contents. Food Chem. (2011) 125:44–8. doi: 10.1016/j.foodchem.2010.08.029

[13] Bag S Mondal A Banik A. Exploring tea (*Camellia sinensis*) microbiome: insights into the functional characteristics and their impact on tea growth promotion. Microbiol Res. (2022) 254:126890. doi: 10.1016/j.micres.2021.126890, 34689100

[14] Lin H Liu C Peng Z Tan B Wang K Liu Z. Distribution pattern of endophytic Bacteria and Fungi in tea plants. Front Microbiol. (2022) 13:872034. doi: 10.3389/fmicb.2022.872034, 36212870 PMC9538792

[15] Karunaratne SHS Abeygunawardena GASI Jayaratne DL Premakumara GAS Chandrasekharan NV. Microbiota of black tea at different manufacturing stages. J Food Saf. (2024) 44:e13152. doi: 10.1111/jfs.13152

[16] Tong W Yu J Wu Q Hu L Tabys D Wang Y . Black tea quality is highly affected during processing by its leaf surface microbiome. J Agric Food Chem. (2021) 69:7115–26. doi: 10.1021/acs.jafc.1c01607 34152762

[17] Liao S Yang S Li B Xia X Jia W Zhao Y . Correlation analysis between key volatile compounds and Core functional bacterial community during Sichuan black tea processing. Food Chem X. (2024) 24:101969. doi: 10.1016/j.fochx.2024.101969, 39582654 PMC11584953

[18] Liao S Mo H Gao Y Bouphun T Xu W Lin L. Study on the influence of microorganisms on the flavor of black tea. Food Chem X. (2025) 31:103173. doi: 10.1016/j.fochx.2025.103173, 41158150 PMC12554971

[19] Bian X Miao W Zhao M Zhao Y Xiao Y Li N . Microbiota drive insoluble polysaccharides utilization via microbiome-metabolome interplay during Pu-erh tea fermentation. Food Chem. (2022) 377:132007. doi: 10.1016/j.foodchem.2021.132007, 34999465

[20] Xia F Hu S Zheng X Wang M Zhang C Wu Z . New insights into metabolomics profile generation in fermented tea: the relevance of bacteria and metabolites in Fuzhuan brick tea. J Sci Food Agric. (2022) 102:350–9. doi: 10.1002/jsfa.11365, 34143449

[21] Dai W Qi D Yang T Lv H Guo L Zhang Y . Nontargeted analysis using ultraperformance liquid chromatography–quadrupole time-of-flight mass spectrometry uncovers the effects of harvest season on the metabolites and taste quality of tea (*Camellia sinensis* L.). J Agric Food Chem. (2015) 63:9869–78. doi: 10.1021/acs.jafc.5b03967, 26494158

[22] Sun Q Wu F Wu W Dong F Huang X Li J . Metabolomics combined with sensory evaluation discriminate Gougunao tea from different geographic origins. LWT. (2025) 229:118196. doi: 10.1016/j.lwt.2025.118196

[23] Yu X Li Y He C Zhou J Chen Y Yu Z . Nonvolatile metabolism in postharvest tea (*Camellia sinensis* L.) leaves: effects of different withering treatments on nonvolatile metabolites, gene expression levels, and enzyme activity. Food Chem. (2020) 327:126992. doi: 10.1016/j.foodchem.2020.126992, 32447133

[24] Jiangxi Administration for Market Regulation. Technical Specification for Processing Gougunao Black Tea; DB36/T 1524—2021. Jiangxi: Jiangxi Provincial Local Standard (2021) (In Chinese).

[25] Wang Q Shi D Hu J Tang J Zhou X Wang L . Unraveling the dynamic changes of volatile compounds during the rolling process of congou black tea via GC-E-nose and GC–MS. Front Sustain Food Syst. (2024) 8:1436542. doi: 10.3389/fsufs.2024.1436542

[26] Dong H Li Y Lai X Hao M Sun L Li Q . Effects of fermentation duration on the flavour quality of large leaf black tea based on metabolomics. Food Chem. (2024) 444:138680. doi: 10.1016/j.foodchem.2024.138680, 38325077

[27] Zhou J Qin M Zhu J Ntezimana B Jiang X Zhang D . Analysis of changes in flavor characteristics of congou black tea at different fermentation degrees under industrial production conditions using flavor compound weighted network co-expression method. Food Chem. (2025) 468:142241. doi: 10.1016/j.foodchem.2024.142241, 39689488

[28] Smith CA Want EJ O’Maille G Abagyan R Siuzdak G. XCMS: processing mass spectrometry data for metabolite profiling using nonlinear peak alignment, matching, and identification. Anal Chem. (2006) 78:779–87. doi: 10.1021/ac051437y, 16448051

[29] Benjamini Y Hochberg Y. Controlling the false discovery rate: a practical and powerful approach to multiple testing. J R Stat Soc Ser B (Stat Methodol). (1995) 57:289–300. doi: 10.1111/j.2517-6161.1995.tb02031.x

[30] Schymanski EL Jeon J Gulde R Fenner K Ruff M Singer HP . Identifying small molecules via high resolution mass spectrometry: communicating confidence. Environ Sci Technol. (2014) 48:2097–8. doi: 10.1021/es5002105, 24476540

[31] Xue J Liu P Yin J Wang W Zhang J Wang W . Dynamic changes in volatile compounds of shaken black tea during its manufacture by GC × GC–TOFMS and multivariate data analysis. Foods. (2022) 11:1228. doi: 10.3390/foods11091228, 35563951 PMC9102106

[32] Bolyen E Rideout JR Dillon MR Bokulich NA Abnet CC Al-Ghalith GA . Reproducible, interactive, scalable and extensible microbiome data science using QIIME 2. Nat Biotechnol. (2019) 37:852–7. doi: 10.1038/s41587-019-0209-9, 31341288 PMC7015180

[33] Callahan BJ McMurdie PJ Rosen MJ Han AW Johnson AJA Holmes SP. DADA2: high-resolution sample inference from Illumina amplicon data. Nat Methods. (2016) 13:581–3. doi: 10.1038/nmeth.3869, 27214047 PMC4927377

[34] Quast C Pruesse E Yilmaz P Gerken J Schweer T Yarza P . The SILVA ribosomal RNA gene database project: improved data processing and web-based tools. Nucleic Acids Res. (2012) 41:D590–6. doi: 10.1093/nar/gks1219, 23193283 PMC3531112

[35] Nilsson RH Larsson K-H Taylor AFS Bengtsson-Palme J Jeppesen TS Schigel D . The UNITE database for molecular identification of Fungi: handling dark taxa and parallel taxonomic classifications. Nucleic Acids Res. (2019) 47:D259–64. doi: 10.1093/nar/gky1022, 30371820 PMC6324048

[36] Bray JR Curtis JT. An ordination of the upland forest communities of southern Wisconsin. Ecol Monogr. (1957) 27:325–49. doi: 10.2307/1942268

[37] Anderson MJ. A new method for non-parametric multivariate analysis of variance. Austral Ecol. (2001) 26:32–46. doi: 10.1111/j.1442-9993.2001.01070.pp.x

[38] Douglas GM Maffei VJ Zaneveld JR Yurgel SN Brown JR Taylor CM . PICRUSt2 for prediction of metagenome functions. Nat Biotechnol. (2020) 38:685–8. doi: 10.1038/s41587-020-0548-6, 32483366 PMC7365738

[39] Ouyang J Jiang R Xu H Wen S Liu C Liu Y . Insights into the flavor profiles of different grades of Huangpu black tea using sensory histology techniques and metabolomics. Food Chem X. (2024) 23:101600. doi: 10.1016/j.fochx.2024.101600, 39071923 PMC11283085

[40] Standardization Administration of China. Methodology for Sensory Evaluation of Tea; GB/T 23776–2018. Beijing: Standards Press of China (2018) In Chinese.

[41] Gloor GB Macklaim JM Pawlowsky-Glahn V Egozcue JJ. Microbiome datasets are compositional: and this is not optional. Front Microbiol. (2017) 8:2224. doi: 10.3389/fmicb.2017.02224, 29187837 PMC5695134

[42] Kurtz ZD Müller CL Miraldi ER Littman DR Blaser MJ Bonneau R. Sparse and compositionally robust inference of microbial ecological networks. PLoS Comput Biol. (2015) 11:e1004226. doi: 10.1371/journal.pcbi.1004226, 25950956 PMC4423992

[43] Liu C Lin H Wang K Zhang Z Huang J Liu Z. Study on the trend in microbial changes during the fermentation of black tea and its effect on the quality. Foods. (2023) 12:1944. doi: 10.3390/foods12101944, 37238765 PMC10217409

[44] Chen Q Fu Y Heng W Yu S Xie F Dong F . Re-rolling treatment in the fermentation process improves the taste and liquor color qualities of black tea. Food Chem X. (2024) 21:101143. doi: 10.1016/j.fochx.2024.101143, 38312489 PMC10837478

[45] Yu Y Chen Z Xie H Feng X Wang Y Xu P. Overhauling the effect of surface sterilization on analysis of endophytes in tea plants. Front Plant Sci. (2022) 13:849658. doi: 10.3389/fpls.2022.849658, 35592578 PMC9111953

[46] Wang X Shan R Li Z Kong X Hou R Wu H . Metabolic improvements of novel microbial fermentation on black tea by Eurotium cristatum. Front Microbiol. (2023) 14:1287802. doi: 10.3389/fmicb.2023.1287802, 38149271 PMC10750952

[47] Friedman J Alm EJ. Inferring correlation networks from genomic survey data. PLoS Comput Biol. (2012) 8:e1002687. doi: 10.1371/journal.pcbi.1002687, 23028285 PMC3447976

[48] Zhou X Tian D Zhou H Dong R Ma C Ren L . Effects of different fermentation methods on flavor quality of Liupao tea using GC-Q-TOF-MS and electronic nose analyses. Foods. (2024) 13:2595. doi: 10.3390/foods13162595, 39200522 PMC11353607

