# Supplementary material for: Comparative profiling of surface-sterilized leaf-associated microbiota and flavor-associated metabolites in two Gougunao tea cultivars across fresh and fermented leaf stages
Source: Front Nutr. 2026 Sep 1;13:1859865. doi: 10.3389/fnut.2026.1859865 (PMC13576263; doi:10.3389/fnut.2026.1859865)
Supplement: Supplementary file 1 [file Supplementary_file_1.DOCX]

**Supplementary Figures**

Supplementary Figure . Global fungal co-occurrence network annotated at the phylum level. Nodes represent fungal genera, including unclassified lineages, and node colours denote phylum-level classification. Edges represent significant Spearman correlations among genera.
